# Comparison of three methodologies for measuring intraocular pressure in healthy cats

**DOI:** 10.14202/vetworld.2024.1803-1809

**Published:** 2024-08-20

**Authors:** Claudia Lizandra Ricci, João Victor Goulart Consoni Passareli, Felipe Franco Nascimento, Glaucia Prada Kanashiro, Luís Felipe da Costa Zulim, Rogério Giuffrida, Silvia Franco Andrade

**Affiliations:** 1 Postgraduate Program in Animal Science, UNOESTE, Presidente Prudente, São Paulo, Brazil; 2 Department of Veterinary Ophthalmology, Veterinary Hospital, UNOESTE, Presidente Prudente, São Paulo, Brazil

**Keywords:** applanation tonometry, goldmann tonometry, intraocular pressure, ocular manometry, rebound tonometry

## Abstract

**Background and Aim:**

Measuring intraocular pressure (IOP) is crucial for identifying potentially damaging changes in the eyes, including diseases as glaucoma and uveitis. This study compared intraocular pressure (IOP) measurements in cats using the Tonovet and Tonovet Plus (rebound), Tono-Pen Avia Vet (applanation), and Kowa HA-2 (Goldman’s methodology applanation) tonometers.

**Materials and Methods:**

55 healthy cats (108 eyes) were assessed through three distinct studies: An *ex vivo* experiment (10 eyes of five cats) to correlate IOP manometry and tonometry values and ascertain the correlation coefficient (r2); an *in vivo* study (10 eyes of five sedated cats) to contrast manometer and tonometer readings; and an outpatient clinical trial (80 eyes of 45 cats) to analyze only tonometer measurements.

**Results:**

The *r*^2^ values observed in the *ex vivo* study were Tonovet (0.923), Tonovet Plus (0.925), Tono-Pen Avia Vet (0.877), and Kowa HA-2 (0.901). The IOP values in mmHg in the *in vivo* study were as follows: Manometer (16.1 ± 2.7), Tonovet (21.1 ± 3.6), Tonovet Plus (19.7 ± 7.2), Tono-Pen Avia Vet (17.6 ± 7.9), and Kowa HA-2 (16.8 ± 2.0). In the outpatient clinical study, the IOP values in mmHg were as follows: Tonovet (19.7 ± 6.6), Tonovet Plus (17.1 ± 5.4), Tono-Pen Avia Vet (16.3 ± 4.3), and Kowa HA-2 (14.5 ± 2.2).

**Conclusion:**

IOP and manometry readings were strongly correlated by all tonometers. In the clinical setting, the most and least IOP measurements were recorded using Tonovet and Kowa HA-2, respectively, stressing the importance of an IOP reference table for each tonometer in feline practice.

## Introduction

Measuring intraocular pressure (IOP) is crucial for identifying potentially damaging changes in the eyes, including high IOP in glaucoma and low IOP in uveitis cases. In contrast to canine glaucoma, feline glaucoma tends to be secondary. Uveitis, a major eye condition, frequently affects domestic cats [1, 2]. The most precise IOP measurement is achieved through invasive ocular manometry [3, 4]. During routine ophthalmic examinations, IOP is measured using tonometry [5–7]. The Tono-Pen (Reichert, USA) applanation tonometer and rebound methodology, with Tonovet and Tonovet Plus (Icare, Finland), are the most common techniques for measuring IOP in cats. In veterinary ophthalmological clinics, applanation tonometry is a widely used, reliable technique in cats. Applanation tonometry can be uncomfortable for animals, necessitating topical anesthesia and direct corneal contact; it demands veterinary expertise to perform correctly and attain accurate readings and can be more time-consuming with increased training requirements. Rebound tonometry, a non-invasive method that does not touch the feline’s cornea or require anesthesia, is easier, quicker, and safer for the cat compared to traditional applanation tonometry. The measurement results from rebound tonometry may exhibit slight variations [1, 2]. In humans, the Goldman applanation method is the customary approach compared to its less frequent usage in animals. Studies have confirmed the accuracy of the Goldman applanation method in cats using Perkins and Kowa HA-2 tonometers [8, 9]. The measurement-recovery principle underpins rebound tonometry, utilizing a magnetized probe that briefly touches the cornea. The software measured the deceleration and duration of contact between the probe and the cornea. Measurements were taken using a disposable probe, without applying topical anesthesia. Applanation tonometry is based on the force required to flatten a given area of a sphere, which is equal to the pressure inside the sphere [4]. Goldmann applanation tonometry uses a 3.06-mm-diameter prism, which measures IOP by the formation of fluorescein semicircles that adjust during the examination [6, 8–10].

In cats, the significance of using different tonometers lies in achieving accurate IOP measurements. Employing multiple tonometers with varying accuracy and methodologies improves the overall accuracy and reliability of IOP assessment in cats. Differences in tonometer usage can enhance our comprehension of feline eye health and drug treatment efficacy. In cats, no comparative study exists on the Goldmann tonometer’s applanation and rebound methods. This study compared and analyzed the use of Tonovet, Tonovet Plus, Tono-Pen Avia Vet, and Kowa HA-2 tonometers in cats.

## Materials and Methods

### Ethical approval

All procedures were approved by the Animal Care and Use Committee of UNOESTE (Protocol No. 4979) and conducted in accordance with the Association for Research in Vision and Ophthalmology guidelines for the use of animals in ophthalmic and visual research. Written informed consent was obtained from the owners or legal custodians of all animals described in this study (experimental or non-experimental animals, including cadavers) for all procedures.

### Study period and location

The study was conducted from March 2019 to March 2022 at the Veterinary Hospital of UNOESTE, Presidente Prudente, São Paulo, Brazil.

### Animals and study design

Fifty-five healthy cats (108 eyes of 28 males and 27 females) of various breeds (one Maine Coon, five Siamese, six Persian, three domestic long-haired, and 40 domestic short-haired) with no signs of eye disease were randomly selected. The animals were considered healthy after physical and ophthalmic examinations, complete blood counts, and liver and kidney function tests. All eyes included in the study underwent slit-lamp examination (SL-15; Kowa), indirect ophthalmoscopy (Pocket Jr; Welch Allyn), Schirmer’s tear test (Ophthalmos), and Fluorescein Test (Ophthalmos) to rule out any ophthalmic conditions affecting the IOP or ocular surface. To standardize the IOP measurement sites, the devices were positioned in the central corneal region and at a 90° angle. Measurements were performed between 1 and 5 p.m. Three IOP readings were recorded, and the average was calculated starting with the left eye and then the right eye, with each tonometer in the following order: Tonovet – “d” calibration, Tonovet Plus – “cat” calibration, Tono-Pen Avia Vet, and Kowa HA-2. IOP measurements were obtained by the same examiner (author CLR) for the TonoVet (Icare), TonoVet Plus (Icare), and Tono-Pen Avia Vet (Reichert) and author SFA for the Kowa HA-2 (Kowa, Japan).

### Procedures

#### Ex vivo study

**Figure-1:**
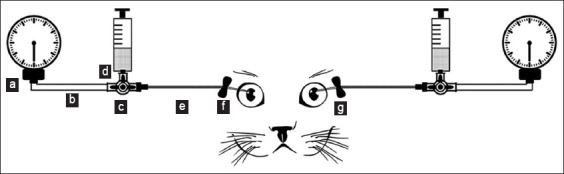
Schematic diagram ex vivo study: (a) Aneroid manometer positioned at the same height in relation to the center of the eye, (b) connected to a polyethylene tube, (c) connected to a three-way stopcock, (d) syringe with a saline solution reservoir, (e) cannulated anterior chamber with a 23G scalp, (f) cannulated at 2 mm posterior to the temporal limbus at 10 o’clock in the right eye, (g) cannulated at 2 o’clock in the left eye.

An *ex vivo* study was conducted to compare the true IOP values obtained by direct ocular manometry with the IOP values measured using tonometers. This methodology was based on previously published studies by Andrade *et al*. [8], Ricci *et al*. [9], Passareli *et al*. [10], McLellan *et al*. [11], and Andrade *et al*. [12]. The study was performed up to 24 h after death in ten healthy eyes of five cats (three males and two females), aged 48–120 months, weight 3.5 ± 1.1 (2–5) kg, at the Veterinary Hospital of UNOESTE. The cats died from causes without ophthalmic repercussions not related to this study. The cats were positioned in the stern recumbency position. The eyelids were separated using a Barraquer blepharostat [13], and the anterior chamber was used with a 23-G scalpel (Lamedid, Brazil) 2 mm posterior to the superior temporal limbus at 10 o’clock position in the right eye and medial superior at 2 o’clock position in the left eye (Figure-1). Cyanoacrylate (Superbonder; Loctite, USA) was applied around the needle to prevent leakage of the aqueous humor. The needle was connected to a polyethylene tube, which was connected to a three-way stopcock (Labor Import) to allow connection to another polyethylene tube and a 0.9% saline solution reservoir (Equiplex, Brazil). An aneroid manometer (BIC, São Paulo, Brazil) attached to this system was zeroed relative to the center of the eye (Figure-1). We artificially raised the IOP by opening a three-way stopcock to infuse saline at 5 in 5 mmHg up to 60 mmHg (10–60 mmHg). Three readings were taken at each IOP level using a tonometer, and the average was calculated.

### *In vivo* study

An *in vivo* study was performed in anesthetized cats to compare the true IOP obtained by direct ocular manometry with that measured using tonometers. We used 10 eyes from five cats (two males and three females), aged 24–132 months, weighing 4.5 ± 0.9 (3.8–6.2) kg, from the UNOESTE cattery. In the surgical center of the veterinary hospital, under the supervision of an anesthesiologist, the anesthetic protocol was based on previously published study by Andrade *et al*. [12] as follows: Pre-anesthetic medication with acepromazine (Acepran 0.2%; Vetnil, Brazil) at a dose of 0.05 mg/kg intravenous (IV) followed by induction with propofol (Propovan; Cristalia, Brazil) at a dose of 5 mg/kg IV, followed by endotracheal intubation and anesthetic maintenance with isoflurane (Isoflurane; Biochimico, Brazil) diluted in 100% O_2_ in a semi-closed circuit. The animals were maintained on artificial ventilation (674 Takaoka Ventilator; KTK, Brazil), and the ventilation parameters were adjusted to maintain an end-tidal carbon dioxide concentration between 35 and 45 mmHg. To centralize the eyeball, the neuromuscular blocker atracurium besylate (10 mg/mL; Tracur, Cristalia) was administered intravenously at a dose of 0.1 mg/kg.

The animals were positioned in sternal recumbency using tonometry with Tonovet, followed by Tonovet Plus. The corneas of both eyes were topically anesthetized with one drop of 1% tetracaine hydrochloride + 0.1% phenylephrine hydrochloride eyedrops (Anestésico; Allergan, Brazil) to measure IOP with Tono-Pen Avia Vet, and then, fluorescein eye drops (Fluoresceína; Allergan) were instilled in the corneas of both eyes to visualize the semicircles in the measurement with Kowa HA-2. After tonometry, ocular manometry was performed to determine true IOP, after which the needle was removed from the anterior chamber and cyanoacrylate glue was instilled with a 1-mL syringe and a 25 × 0.7-mm needle (BD, São Paulo, Brazil) at the corneal puncture site to seal the perforation. Subsequently, to the IOP readings, the effects of atracurium besylate were reversed with the use of neostigmine methylsulfate (Normastig; União Química, Brazil) at 0.5 mg/mL at a dose of 0.01–0.04 mg/kg and atropine sulfate at 0.25 mg/mL at a dose of 0.044 mg/kg (Pasmodex; Isofarma, Brazil). After this procedure, the animals were treated with one drop every 8 h for 7 days with tobramycin antibiotic eye drops (Tobrex; Novartis, Brazil) and diclofenac sodium 0.1% anti-inflammatory eye drops (Still; Allergan) and assessed by daily ophthalmic examination.

### Outpatient clinical study

**Figure-2:**
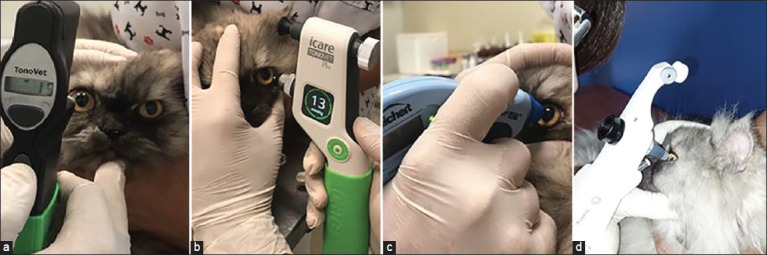
Outpatient clinical study with the tonometers used for intraocular pressure readings: (a) Tonovet, (b) Tonovet Plus, (c) Tono-Pen Avia Vet, and (d) Kowa HA-2.

To evaluate the use of tonometers in routine clinical practice, IOP was measured in the eyes of cats treated at an outpatient clinic in the Ophthalmology Department of a Veterinary Hospital (UNOESTE). A total of 45 cats (88 eyes) were evaluated, of which two animals had only one eye as a result of post-traumatic enucleation of the contralateral eye, age between 1.5 and 180 months, weight 3.7 ± 1.6 (0.4–7) kg, 23 males and 22 females. The cats were positioned in a station, their neck was kept without pressure, and their eyelids were slightly turned apart to avoid changes in the IOP measurement, and a 2-min interval [10] was maintained between the use of each tonometer (Figure-2).

### Statistical analysis

In the *ex vivo* study, regression lines were constructed to measure the IOP values from manometry *versus* tonometry and calculated the correlation coefficient (*r*^2^) and the linear regression equation. The Bland–Altman agreement analysis was performed to compare two quantitative methods of measuring IOP and to define a series of agreements as a mean bias of ± 2 standard deviations. In the *in vivo* and outpatient clinical studies, the mean and standard deviation of the IOP values measured using tonometers were calculated and statistically compared using analysis of variance. Statistical significance was set at p < 0.05.

## Results

**Figure-3:**
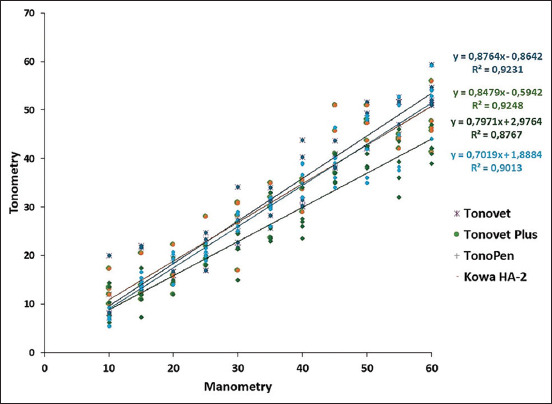
Comparison of intraocular pressure measurements in mmHg between manometry (aneroid manometer) and tonometry (Tonovet, Tonovet Plus, Tono-Pen Avia Vet, and Kowa HA-2) in five cats (n = 10 eyes) in an *ex vivo* study. The solid line is the calculated regression line. *r*^2^ (correlation coefficient).

**Figure-4:**
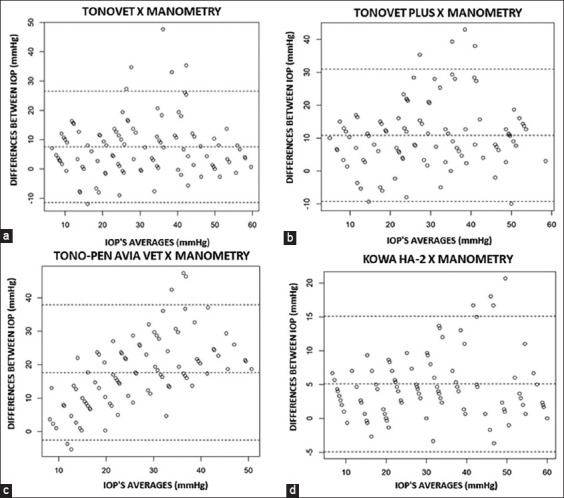
Bland–Altman plot comparing intraocular pressure in mmHg in five cats (n = 10 eyes): (a) Tonovet tonometer and manometer, (b) Tonovet Plus tonometer and manometer, (c) Tono-Pen Avia Vet tonometer and manometer, and (d) Kowa HA-2 tonometer and manometer.

In the *ex vivo* study, there was a strong correlation between the manometric IOP values and all tonometers, with the observed values of *r*^2^ in decreasing order: Tonovet Plus (0.925), Tonovet (0.923), Kowa HA-2 (0.901), and Tono-Pen Avia Vet (0.877). The linear regression equations for each tonometer are shown in Figure-3. In the analysis of agreement observed in the Bland-Altman graphs (Figure-4), Tonovet showed a slight tendency to underestimate the manometry IOP values close to 15 mmHg and overestimate IOP readings between 25 and 40 mmHg. Tonovet Plus also showed reduced accuracy in the values measured between 25 and 40 mmHg and underestimated IOP readings close to 50 mmHg. Tono-Pen Avia Vet showed less agreement at low pressures, with the underestimation of the readings between 10 mmHg and 15 mmHg and overestimation of readings above 35 mmHg, and the Kowa HA-2 tonometer overestimated IOP values at pressures above 40 mmHg.

**Figure-5:**
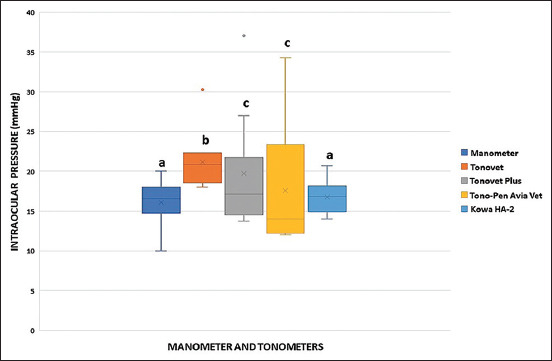
Boxplot of intraocular pressure (mmHg) readings between manometry (aneroid manometer) and tonometry (Tonovet, Tonovet Plus, Tono-Pen Avia Vet, and Kowa HA-2) in 10 normal eyes of five cats in the *in vivo* study. Different letters indicate significant differences (p < 0.05). •: outlier.

In the *in vivo* study, the IOP values measured using manometry and different tonometers are shown in Figure-5. There were significant differences (p < 0.05) between manometry and the Tonovet, Tonovet Plus, and Tono Pen Avia Vet tonometers but not between manometry and the Kowa HA-2 tonometer (p > 0.05) (Figure-5).

**Figure-6:**
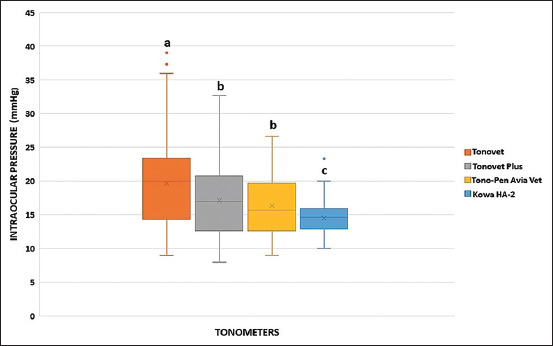
Boxplot of intraocular pressure (mmHg) readings from tonometers (Tonovet, Tonovet Plus, Tono-Pen Avia Vet, and Kowa HA-2) in 88 normal eyes of 45 healthy cats, evaluated in the outpatient clinical study. Different letters indicate significant differences (p < 0.05). •: outlier.

In the outpatient clinical study, there was a significant difference (p < 0.05) between Tonovet and the other tonometers; however, no significant difference was observed between Tonovet Plus and Tono-Pen Avia Vet. Tonovet and Kowa HA-2 presented the greatest and smallest variations in IOP values, respectively, compared with the other tonometers (Figure-6).

## Discussion

This is the first study in the world to compare applanation tonometry using the Goldmann methodology (Kowa HA-2) with two other methodologies frequently used by veterinary ophthalmologists: applanation (Tono-Pen Avia Vet) and rebound (Tonovet and Tonovet Plus) in cats. Although applanation tonometry using the Goldmann methodology is rarely used in veterinary medicine, some studies have shown an excellent correlation between manometry and the Perkins [8, 12] and Kowa HA-2 [9] tonometers in cats. A vast body of literature is available on the use of applanation tonometers with Tono-Pen Vet [13–17] and Tono-Pen XL [12, 18–20] and with the rebound methodology tonometer Tonovet [11, 14, 16, 17, 21] in cats; however, only one study reported the applanation methodology with Tono-Pen Avia Vet [22], and two studies reported the rebound methodology with Tonovet Plus [14, 23].

In the *ex vivo* study, there was a strong correlation (*r*^2^ > 0.8) between manometry and tonometers in the following decreasing order: Tonovet Plus (0.925), Tonovet (0.923), Kowa HA-2 (0.901), and Tono-Pen Avia Vet (0.877). These values are similar to those of a recently published study on dogs using the same device [10]. Rusanen *et al*. [17] compared Tonovet tonometers with Tono-Pen Vet in cats and concluded that both were correlated with direct manometry. McLellan *et al*. [11] conducted a comparative study of Tonovet and Tono-Pen XL in cats and found that Tonovet was significantly more accurate than Tono-Pen XL, in agreement with our results.

In the Bland-Altman agreement analysis, the two rebound tonometers evaluated in this study (Tonovet and Tonovet Plus) overestimated IOP readings between 25 and 40 mmHg but correlated well with direct manometry, with the best agreement in the 45–60 mmHg range. This contradicts a validation study carried out by Tonovet in cats, which demonstrated an overestimation of IOP at higher pressures and an increase in variance, particularly between 40 and 60 mmHg [11]. However, in another comparative study with Tonovet in cats, a good correlation with direct manometry was observed, with the best agreement in the 25–50 mmHg range, despite the tonometer underestimating IOPs below 25 mmHg and showing reduced accuracy only at pressures above 50 mmHg [17].

An experimental study conducted using the Tono-Pen XL tonometer observed that this tonometer underestimated IOP values between 10 and 50 mmHg in cats [20]. In another study conducted using the same tonometer, an underestimation of 3–5 mmHg was observed in all IOP measurements in cats [11]. In this study, the Tono-Pen Avia Vet underestimated lower IOP values and overestimated IOP values at higher pressures, particularly between 35 and 40 mmHg. Better agreement was found for readings between 20 and 30 mmHg in cats in this study, which contradicts the findings of Martinez and Plummer [14] that Tono-Pen Vet was consistent with manometry closer to 10 mmHg in dogs and cats.

Goldmann applanation tonometry with the Kowa HA-2 correlated well with direct manometry in our study and showed the best agreement for IOP readings between 10 and 40 mmHg. In addition, the Kowa HA-2 IOP readings were closest to those of ocular manometry in the *in vivo* evaluation. Existing studies on Goldmann tonometry in cats using the Perkins tonometer [8, 12, 24] and Kowa HA-2 [9] have reported similar results.

Knowledge of the differences between tonometers can help clinicians interpret IOP values obtained using different devices [25]. In the clinical evaluation, Tonovet measured significantly higher IOP values (p > 0.05), with average results of 3–6 mmHg above the values recorded by the other tonometers, similar to the findings of Rusanen *et al*. [17], who observed average IOP values between 2 and 3 mmHg above those measured with the Tono-Pen Vet applanation tonometer. Similar to our findings, in a comparative study of the same four tonometers in dogs [10], a significant difference was observed. However, with Tonovet Plus, readings were between 3 and 5 mmHg higher than those of the other tonometers, which were also corroborated by a similar study comparing Tonovet, Tonovet Plus, and Tono-Pen Avia Vet in dogs with normal eyes, where the values of Tonovet Plus were also significantly higher than those obtained with Tonovet, which were significantly higher than those obtained with Tono-Pen Avia Vet [25, 26]. In a recent study by Kerdchuchuen *et al*. [23], who compared the use of Tonovet Plus in brachycephalic and non-brachycephalic cats, IOP values obtained from non-brachycephalic cats (18.77 ± 0.49 mmHg) were similar to those obtained in our study (17.1 ± 5.4 mmHg).

Rebound tonometry is well tolerated by cats [17, 27] and was well tolerated in this study. Despite this, it was observed in our study that the innovations of Tonovet Plus recently launched do not favor its use in cats. The animals were startled by the beeps emitted at each measurement and the signals around the probe. It is recommended that these functions should be deactivated when necessary in clinical practice. In the outpatient clinical study, the IOP values varied between tonometers. The IOP values in mmHg were as follows: Tonovet 19.7 ± 6.6 (9.0–39.0), Tonovet Plus 17.1 ± 5.4 (8.0–32.7), Tono Pen Avia Vet 16.3 ± 4.3 (9.0–26.7), and Kowa HA-2 14.5 ± 2.2 (10.0–23.6). High IOP values with Tonovet and low and less variable values in Kowa HA-2 were close to those reported in cat studies by Tonovet [17] and Kowa HA-2 [9]. The Tonovet and Tonovet Plus rebound tonometers were the easiest and fastest to use for measuring IOP. The characteristics of the cats observed in this study using tonometers are shown in Table-1.

**Table-1:** Characteristics observed by the authors in this study using the Tonovet, Tonovet Plus, Tono-Pen Avia Vet, and Kowa HA-2 tonometers in the clinical evaluation of healthy cats without ocular alterations.

Characteristics	Tonovet	Tonovet Plus	Tono-Pen Avia Vet	Kowa HA-2
IOP values (mmHg)	16–25	15–24	15–25	12–18
Accuracy	+++	+++	++	+++
Acceptance by cats	+++	++	++	++
Ease of use	+++	+++	++	+
Disposable probe	Yes	Yes	Yes	No
Topical anesthesia	No	No	Yes	Yes

+=Low, ++=Moderate, +++=High, IOP=Intraocular pressure

This study was limited by its small sample size compared with that used in tonometry studies in dogs, such as those by Passareli *et al*. (n = 76) [10] and von Spiessen *et al*. (n = 80) [28]. However, the number of cat eyes used in the present *ex vivo* (n = 10) and *in vivo* (n = 10) studies for the correlation of manometry and tonometry are greater than those reported by Rusanen *et al*. (n = 6) [17] and McLellan *et al*. [11], who used one normal cat (n = 2 eyes) and two cats with glaucoma (n = 4 eyes), and Martinez and Plummer (n = 6 eyes) [14].

## Conclusion

There was a strong correlation between the IOP values obtained using manometry and those obtained using the TonoVet, TonoVet Plus, Tono-Pen Avia Vet, and Kowa HA-2, demonstrating the high accuracy of these tonometers in healthy cats with normal eyes. The Kowa HA-2 tonometer showed that the IOP readings approached manometry *in vivo*, with smaller variations. In a clinical outpatient study, the highest and lowest IOP values were measured using Tonovet and Kowa HA-2, respectively. Therefore, the current study provides more information on the use of these devices in cats and reinforces the need for a table of IOP reference values for each tonometer.

## Authors’ Contributions

CLR: Designed and conducted the experiments, performed all animal examinations and tests, analyzed the data, prepared graphs, figures, and tables, and drafted the manuscript. JVGCP, LFCZ, and FFN: Performed animal examinations. GPK: Anesthetized and monitored the animals during the *in vivo* study. RG: Designed and analyzed the data and prepared the graphs and tables. SFA: Conceptualized the aim of the study, planned, supervised, designed, and conducted the experiment, performed animal examinations and tests, analyzed the data, and drafted and revised the manuscript. All authors have read, reviewed, and approved the final version of the manuscript.
